# Periarticular infiltration used in total joint replacements: an update and review article

**DOI:** 10.1186/s13018-023-04333-z

**Published:** 2023-11-13

**Authors:** Gavin Anthony King, Alexander Le, Michaela Nickol, Bianca Sarkis, Johannes Michiel van der Merwe

**Affiliations:** 1 Present Address: College of Medicine - Department of Orthopaedic Surgery, University of Saskatchewan, Saskatoon, Canada; 2 Faculty of Medicine - Division of Orthopaedic Surgery, University of Montreal, Montreal, Canada

## Abstract

Periarticular infiltration following total knee and hip arthroplasty has been demonstrated to be equivalent to peripheral nerve blocks for postoperative pain management. The ideal cocktail has not been established yet. We have conducted a literature search on PubMed and Embase. Our search criteria included randomized controlled trials (RCTs) and systematic reviews (SRs). We tried to only include the most recent studies to keep the information current. The included research focused at Dexmedetomidine, Liposomal Bupivacaine, Ropivacaine, Epinephrine, Ketorolac, Morphine, Ketamine and Glucocorticosteroids. Each medication’s mode of action, duration, ideal dosage, contraindications, side effects and effectiveness have been summarized in the review article. This article will help the clinician to make an informed evidence-based decision about which medications to include in their ideal cocktail.

## Introduction

Periarticular infiltration is an effective adjunct to multimodal pain management following a joint replacement. Multiple drugs have been proposed to use in the periarticular cocktail. Unfortunately, there is no consensus on which medications to use. This review article summarizes the most recent evidence available for the most commonly used medicines in periarticular blocks. In addition, there are multiple sites proposed for injection. Ross et al. have summarized the best potential locations for periarticular injection based on nociceptor prevalence [1]. The highest concentrations of nociceptors in the knee were found in the medial and lateral retinacula, infrapatellar fat pad, pes anserine bursa, tibial, femoral, and patellar periosteum, and the bony insertions of the MCL, LCL and IT band [1]. In the hip articulation, there is limited research, but higher concentrations of nociceptors have been found in the labral base, ligamentum teres and the hip capsule [1]. No data exist regarding nociceptor concentration for tendon insertions around the hip joint.

We have conducted a literature search on PubMed and Embase. Our search criteria included randomized controlled trials (RCTs) and systematic reviews (SR). We tried only to include the most recent studies to keep the information current. We included only studies published in the English language. We aimed to have at least ten studies available for each drug. If insufficient RCTs and systematic reviews (SRs) were available, we broadened the search to include other specialties that also utilize local infiltration following surgery. Our search terms included: dexmedetomidine; Presedex, liposomal ketorolac, ketorolac, Toradol, tranexamic acid, morphine, glucocorticosteroids, triamcinolone acetate, methylprednisolone, betamethasone, ropivacaine, epinephrine, total knee arthroplasty, total knee replacement, periarticular infiltration, local anesthetic, peripheral block in combination with Boolean operators.

## Dexmedetomidine (Presedex)

Using the search criteria, we identified 12 randomized controlled trials evaluating dexmedetomidine (Table 1) [2–13]. Dexmedetomidine (DM) is a highly selective alpha-2 adrenoceptor agonist with a half-life of 2 h [2]. It binds an alpha-2 receptor eight times more than clonidine [3]. Though multiple theories have been proposed, the exact mechanism of action is not completely understood. The first prevalent theory suggests that DM activates the alpha-2 adrenoreceptors on the peripheral smooth muscle cells, leading to vasoconstriction with subsequent delayed absorption of the local anesthetic [2]. Secondly, it was proposed that DM can block the activity-dependent cation current [2]. The activation of the Na–K pump causes hyperpolarization of the membrane in the peripheral fibers. DM could enhance hyperpolarization by blocking the Na–K currents, thus inhibiting the action potentials [7].

**Table 1:** Articles included in the review analyzing the use of dexmedetomidine in periarticular infiltrations

Name	Type of study	Number of patients	Groups and findings	Dosage of DM	Duration of DM	Main findings	Complications (postop delirium, Nausea, Hypotension, Bradycardia	Exclusion criteria
Zhao et al. [2]	RCT	116 total knee arthroplasty patients	Control (Ropivicaine) (1) Epinephrine + Ropivicaine (2) Dexmedetomidine + Ropivicaine(3) DM had lower pain scores at 6 h DM + Epinephrine had lower pain scores for first 24 h	2.0 ug/kg	Good pain relief 6–12 h after surgery	Lower levels of proinflammatory cytokines. Prolong sensory block and minimize motor block effect	No complications	Allergies to the medications; severe knee deformities;history of a knee infection;Long-term opioid usage; history of neuromuscular disorders;diagnosis of severe cardiovascular disease
Nickar et al. [3]	RCT	75 total knee arthroplasty patients	DM + Ropivicaine(1) Ketorolac + Ropivicaine (2) Control (Ropivicaine) (3)	1.0 ug/kg	150 min ± 54.22 pain relief	Ketorolac demonstrated better pain relief compared to DM	No complications	Patients with a history of infection or malignant disease; cardiac disease(receiving Beta-Blockers, calcium channel blockers or alpha-methyldopa, coagulopathy, hepatic or kidney disease, allergic reaction to medications
Salem et al. [4]	RCT	45 knee arthroscopy patients	Control (1) DM (2) Fentanyl (3)	100 ug	505 min pain relief	Fentanyl had the longest pain relief (693 min) First 6 h had same pain relief between Fentanyl + DM	No complications	Advanced renal or hepatic disease;psychiatric disorders;prolonged intake of NSAIDS, patients taking beta-blockers-tricyclic antidepressants; allergic reactions to medications
Pangrahi et al. [5]	RCT	60 arthroscopy patients	(1) Control (2) DM (1 ug/kg) (3) DM (2 ug/kg)	1–2 ug/kg	757.3 ± 207.68 min in group 3	Group 3 demonstrated significant lower pain scores in first 12 h, lower analgesic requirement and lower intensity of pain	No complications	Allergy to medications;pregnancy or lactation;hepatic-renal-cardiopulmonary abnormality; alcoholism,diabetes,bleeding diathesis,patients of Beta-blockers,clonidine,alpha-methyldopa; patients using opioid or non-opioid analgesia
Mohamed et al. [6]	RCT	90 abdominal hysterectomy patients	(1) Bupivicaine 0.25% + 2 mg/kg Ketamine (2) Bupivicaine 0.25% + DM 2 ug/kg	2 ug/kg	(1) Ketamine – 7.6 ± 4.16 h (2) DM – 6.00 ± 3.73 h	Rescue analgesia, first dose of analgesia, VAS score was less in Ketamine + DM group with Ketamine doing slightly better than DM	6.7% of patients had sedation + hypotension in DM group	Significant cardiac,respiratory,renal or hepatic diseases, coagulation disorders, patients with infection, patients with psychiatric illnesses
Hao et al. [7]	RCT	120 patients undergoing tonsillectomies or adenoinectomies	(1) GA + Ropivicaine + DM (2) GA + Ropivicaine	1 ug/kg	10 h (9.4–11.4)	Pain scores were significantly lower at the 8^th^,16^th^,20th and 24^th^ hour after surgery	None documented	Patients with cardiovascular,respiratory and endocrine disorders; Patients with known allergies to local anesthetics
Azemati et al. [8]	RCT	60 pediatric patients undergoing unilateral herniorraphy	(1) Bupivicaine 0.5% + DM (2) Bupivicaine 0.5%	1 ug/kg	2 h	Pain scores were better in the first 2 h for the DM group	Heart rate was statistical lower in the DM group in the first 20 min after injection. Sedation was also significantly more in the DM for the first 3 h	Patients with history of seizures, coagulopathies,sensitivity to DM, congenital heart disease, history of bleeding disorders, liver or kidney failure, neurological diseases
Abo Elfadl et al. [9]	RCT	90 children undergoing tonsillectomies	(1) Levobupivicaine 0.25% + DM (2) Levobupivicaine 0.25%	1 ug/kg	644.31 ± 112.89 min	The DM group consumed less analgesia in the first 24 h and had a higher total oral intake	No side effects	Obstructive sleep apnea syndrome;cardiovascular,liver,renal disease,coagulation disorders,relevant drug allergies,neurological or psychiatric illness
Mitra et al. [10]	RCT	45 undergoing elective lumbar discectomies	(1) Ropivicaine 0.5% (2) Ropivicaine 0.5% + Tramadol (3) Ropivicaine 0.5% + DM	0.5 ug/kg	930 min (854.3–1005.7)	DM group demonstrated statistical longer time to rescue analgesia. Pain scores and Diclofenac consumption in the first 24 h was statistically lower in the DM	No side effects	ASA grade ¾;History of allergy to relevant drugs;patients with severe systemic disease,pregnancy,psychiatric illness,seizure disorder
Li et al.[11]	RCT	57 patients with elective posterior lumbar fusion	(1) Ropivicaine 0.5% (2) Ropivicaine 0.5% + DM	1 ug/kg	10.5 ± 3.7 h	Significantly less Morphine + PCA requirements in the DM. Analgesic request was delayed and Vas core reduced up to 16 h after surgery	No side effects	Allergies to relevant medications; long-term treatment with opioids;renal or hepatic insufficiency;neoplastic disorders;BMI > = 30, local sepsis;unbalanced cardiopathy or pneumopathy, severe diabetes;preoperative psychiatric or cognitive disorders,coagulation abnormalities
Yu et al. [12]	RCT	140 patients undergoing laparoscopic cholecystectomy	(1) Placebo group (saline) (2) Ropivacaine 0.5%(skin); + Saline(deltoid muscle) (3) ropivacaine 0.5% + DM (Skin); and Saline at the deltoid muscle (4) Ropivicaine 0.5% (skin); and DM at the deltoid muscle	1 ug/kg	12 h	DM did demonstrate lower Vas score scores compared to Ropivicaine + saline up to 12 h. The Ropivicaine + DM combined at the sking performed the best	No adverse events. Slight decrease in diastolic + systolic blood Pressure in DM groups (no statistical significant)	BMI > = 30; renal or hepatic disorders, opioid usage;history of alcohol or drug abuse;ASA status or 3 or greater;contraindications of use(hypotension or bradycardia);pregnancy, patients with history of chronic pain; allergic to relevant medications
Luan et al. [13]	RCT	50 patients undergoing an open gastrectomy	(1) Ropivicaine 0.3% (2) Ropivicaine 0.3% + DM	1 ug/kg	Didn’t record first time to analgesia	No difference in VAS scores between groups. Higher Sufentanil consumption in the first 24 h	No adverse events	Allergy to relevant medications; BMI > = 30; opioids addiction; chronic pain, psychiatric diseases

Because of the sedating effects, anesthesiologists routinely use intravenous (IV) DM to help sedate patients during a surgical procedure. If DM is used in the periarticular block, the sedating effects can improve sleep at night, a common problem after hip or knee replacements. Additional benefits include: reduced opioid usage, prolonged neuraxial analgesia, decreased postoperative delirium and reduced postoperative nausea [14]. In addition to the sedative effects described above, it also has anxiolytic, analgesic, anesthetic-sparing and sympatholytic effects [3].

Yang et al. [14] demonstrated a dose-dependent response using IV DM and postoperative hypotension and bradycardia. They suggested using the lowest possible dose (< 50ug) to minimize hemodynamic side effects. Even though there was a statistical difference in hemodynamic side effects between patients receiving DM compared to patients not receiving DM, they did not find a statistical difference comparing side effects between different doses of DM (< 50; 50–99; > 100ug). It is important to note that conclusions cannot be extrapolated from IV administration of DM to periarticular infiltration. Fritsch et al. [15] found that blood concentration was extremely low at 180 min after surgery. In this study, 150ug DM was added to the interscalene brachial plexus block [15].

The dosing of DM is not clearly defined, with different dose recommendations per route administration. Typically, IV dosing should be as minimal as possible (< 50ug), while intramuscular (2.5ug/kg) or periarticular dosing (1–2 ug/kg) can be more generous due to the slower absorption and low blood concentrations [2, 15, 16]. In the RCTs in Table 1, the dosages varied between 0.5 and 2 ug/kg. There was no correlation between dosage and duration of analgesia. DM showed a benefit in combination with longer-acting anesthetic drugs, such as ropivacaine and levobupivacaine, demonstrating longer-lasting pain relief [12].

There were minimal complications observed with the addition of DM. Only two studies from the twelve RCTs listed in Table 1 had observed side effects (bradycardia, hypotension and sedation) [6, 8]. Therefore, with careful screening, DM can be a valuable addition to the periarticular block. The most commonly used exclusion criteria in the studies in Table 1 include allergies to the medications [2, 4, 5, 7–13], diagnosis of severe cardiovascular disease [2, 5–12], diagnosis of hepatic or renal disease [2, 4], psychiatric disorders [4, 9–11, 13], pregnancy or lactation [5, 6, 10, 12], and history of neuromuscular disorders [2, 8–10]. Care should be exercised in these groups of patients. It is advisable to discuss patients with these conditions with your anesthesiologist and consider omitting or using smaller doses of DM (e.g., 0.5ug/kg).

## Epinephrine

Epinephrine is commonly used in periarticular injections. It is a sympathomimetic catecholamine that affects alpha- and beta-adrenergic receptors. Its effect on Alpha-1 receptors produces increased vascular smooth muscle contraction. It is thought by this mechanism to create a synergistic effect when used with local anesthetics [17]. Epinephrine is believed to reduce the peripheral blood flow, prolonging the duration of local anesthetics and increasing the maximal dose that can be used without fear of systemic toxicity [17]. Its effect on the duration of local anesthetic is controversial, as it has not been well demonstrated. It is also thought to cause potentially harmful effects on wound healing through the local vasoconstriction at the level of the skin when blood flow is poor [17].

A literature review concerning the use of epinephrine in periarticular blocks does not reveal a clear consensus on its utility (Table 2) [18–24]. Some studies have reported statistically decreased visual analog pain scores when epinephrine is used in the periarticular block [19, 22], but this did not reach clinical significance. Contrarily, Kong et al. [18] showed no significant differences in pain scores postoperatively nor on opioid use after arthroplasty. There is some evidence that epinephrine can reduce postoperative bleeding when used in the periarticular block without increasing deep venous thrombosis (DVT) risk [21, 23]. However, it does not influence intraoperative blood loss, postoperative hemoglobin levels, or postoperative transfusion rates [21, 22].

**Table 2:** Articles included in the review analyzing the use of epinephrine in periarticular infiltrations

Name	Type of study	Number of patients	Groups and findings	Administration	Findings	Complications
Kong et al. [18]	RCT	116 patients (134 knees)	2 randomly allocated groups: Epinephrine mixed in or not	0.3 mL epinephrine (1:1000) mixed with ropivacaine and triamcinolone	No significant differences in postoperative numerical pain scale, dose of fentanyl using PCA or active range of motion	No complications
Chareancholvanich et al. [19]	RCT	80 TKA patients	2 randomly allocated groups: epinephrine, control	20 mL 0.5% bupivacaine, 30 mg ketorolac, ± 0.6 mg epinephrine (1:1000), diluted with NS for a total volume 100 mL	Statistically significant reduction in visual analog pain scores at 6 and and 12 h with reduced morphine hours. Magnitude did not reach MCID for TKA	None
Liu et al. [20]	RCT	195 THA patients	3 groups: intravenous low dose epinephrine + TXA, topical diluted epinephrine plus TXA, TXA alone		Combined LDEPI and TXA was more effective in reducing periop blood loss and alleviating inflammatory response than TXA alone	No complications
Teng et al. [21]	Metanalysis	5 studies in THA/TKA	335 in epinephrine group, 311 in control group	intra-articular, local, lavage or IV	Epinephrine significantly reduced postop bleeding volume in TJA without increasing DVT risk. No reduction in intraop blood loss, postop hemoglobin loss and transfusion rate	No complications
Villatte et al. [22]	RCT	150 THA patient	Group 1: ropivacaine with epinephrine, Group 2: no infiltration		LIA patients have less pain using VAS scores, no differences in analgesic consumption, recovery or bleeding	No complications
Gao et al. [23]	RCT	100 THA patients	Group 1: 3 g intra-articular TXA + 0.25 mg diluted epi. Group 2: 3 g topical TXA alone		Topical TXA plus epinephrine reduced total blood loss, hidden blood loss and transfusion	No complications
Mikjunovikj-Derebanova et al. [24]	RCT	63 pediatric patients undergoing surgical treatment of upper limb fractures	Group 1: Lidocaine 2%, bupivacaine 0.25% to a total volume of 0.5 ml/kg Group 2: 25 mcg epinephrine in 2 mL of 2% solution of lidocaine and 0.25% bupivacaine to a total volume of 0.5 ml/kg Group 3: 2% lidocaine 2 mL and 0.25% bupivacaine with 2 mg dexamethasone to a total volume on 0.5 ml/kg	Supraclavicular or interscalene block with one of three cocktails	Epinephrine prolonged motor and sensory effects for about 30 min on average. Dexamethasone compared to both groups prolonged both sensory and motor effects in a statistically significant manner	None

Dosing of epinephrine as part of a periarticular cocktail does not have clear recommendations for administration. In the reviewed studies, epinephrine was typically diluted at concentrations of 1:1000 for total dosages ranging between 25 and 60 mcg. No studies compared different dosages of epinephrine.

No complications were recorded in any of the reviewed studies regarding the use of epinephrine in periarticular infiltrations. Specifically, no wound-healing problems related to using epinephrine in the periarticular cocktail were recorded.

Therefore, the benefits and recommended use of epinephrine as part of a periarticular block still need to be determined.

### Glucocorticosteroids

Triamcinolone acetonide is a synthetic corticosteroid with a half-life of 18–36 h [25]. The steroids' acetate form is insoluble and postulated to remain in the tissues to give anti-inflammatory properties. This can be potentiated by the vasoconstricting effects of epinephrine [25, 26]. Methylprednisolone is a synthetic glucocorticosteroid with a half-life of 1.8–2.6 h [27, 28]. Betamethasone and dexamethasone have half-lives of 36–54 h [29–31]. All the corticosteroids examined in this review paper are primarily metabolized in the liver and excreted by the kidneys.

Glucocorticosteroids (GCSs) are thought to reduce the stress response, decrease edema and blood loss, prevent nausea and vomiting and achieve a better permanent range of motion by reducing the inflammatory response [32]. Pain is induced by inflammation. Inflammatory markers (IL-1Beta, IL-6, TGF-alpha) are released after surgery, leading to a decrease in the nociceptor threshold with subsequent occurrence of pain. GCS inhibits phospholipase A-2, which reduces the proinflammatory derivatives of arachidonic acid, decreasing inflammation [33, 34]. Interestingly, only one study [26] evaluated the inflammatory markers in patients receiving GCS and found a reduction in the CRP values until postoperative day four compared to the control group.

GCS also leads to decreased production of prostaglandins with their vasodilatory effects and a subsequent diminution in blood loss [33, 34]. Again, only two studies from the RCTs listed evaluated blood loss in patients receiving GCS as part of the PAI and found a non-statistical decrease in blood loss in the GCS group [26, 33].

There is, however, hesitancy in administering GCS routinely after joint replacements because of a fear of poor wound healing, postoperative infections or ligament/tendon ruptures. When evaluating the injection sites in the PAI, most studies injected GCS as part of the cocktail into the MCL [25–28, 30, 31, 35, 36] and LCL [26–28, 30, 31, 35, 36]. Four studies injected GCS into the patella tendon and fat pad [27, 28, 31, 35], while others did not inject GCS into the fat pad or patella tendon in fear of late rupture [25, 32, 36]. Two studies did not inject GCS in the subcutaneous skin to avoid steroid-induced skin atrophy [31, 33].

Most studies excluded patients with a history of renal insufficiency, uncontrolled diabetes mellitus, local infection, history of cardiac arrhythmias or prolonged QT intervals, immunosuppression (e.g., inflammatory conditions), history of congestive heart failure, psychiatric illnesses or history of gastrointestinal bleeding [25–28, 30, 31, 33, 35, 36].

No clear conclusions can be derived from the results of the randomized controlled trials in Table 3 [25–33, 35, 36]. Multiple studies demonstrated lower VAS scores in the immediate postoperative period. Most studies showed an effect in the 12–24-h postoperative period [26–28, 30, 32, 33, 35], while longer duration up to 48 h is supported only by several studies [27, 28, 33]. 72 h or more of lower VAS scores were only observed in a few studies [28, 33]. Even though some studies did not report any difference in VAS scores with the addition of corticosteroids [25, 31, 36], it does seem plausible to assume GCS can improve pain in the early postoperative period.

**Table 3:** Articles included in the review analyzing the use of glucocorticosteroids in periarticular infiltrations

Name	Type of study	Patients	Groups	Main findings	Complications	Exclusion criteria
Reddy et al. [27]	Prospective randomized controlled trial	140 patients MP group 70 patients Non steroid group—70 patients	Periarticular cocktail (PAI) 60 ml of 0.2% Ropivacaine; 8 mg Morphine; 0.3 mg Epinephrine; 60 mg Ketorolac MP group received an additional 100 mg of MP while the second group received only Saline	Lower VAS score in PAI group with Methylprednisolone 8 h after surgery, POD 1 and POD 2; No difference in ROM between the groups	Not listed	Bilateral of revision total knee arthroplasty procedures; General or Epidural anesthesia; severe renal insufficiency; uncontrolled DM, Bleeding disorders; history of vascular surgery involving the femoral vessels on operative side; History of arrhythmias and seizures; History of chronic pain, or alcohol or drug abuse, Allergies to medication, local infection, sepsis
Kulkarni et al. [28]	Randomized controlled trial	50 patients undergoing bilateral TKA	Both knees received a PAI, but the second knee also received methylprednisolone (40 mg) as part of the PAI	Lower VAS at 24 h and 72 h in the PAI with Methylprednisolone group. Greater ROM at 24 h and 72 h after surgery in the PAI with methylprednisolone group	No infections or further operations in the subsequent 6 months	Renal insufficiency; poorly controlled DM (HBA1C > 7.0); history of inflammatory arthritis;Allergy to medication; history of knee infection or prolonged QT interval
Chia et al. [25]	Randomized Controlled trial	127 patients	3 groups. One control group, 1 low dose steroid group (40 mg Triamcinolone acetonide) and 1 high dose steroid (80 mg) group	No significant differences between pain scores and ROM between the groups	One PJI in the high dose steroid group	Unstable Diabetes, Immunosuppression,Chronic renal failure, Allergic reaction
El-Boghdadly et al. [29]	Randomized controlled trial	140 patients 72 patients—saline group 68 patients—Dexamethasone group	PAI consisted of 300 mg Ropivicaine, 30 mg Ketorolac; 0.6 mg of Epinephrine Patients randomized to receive 8 mg of Dexamethasone or Saline	Dexamethasone group had lower rates of Nausea + Vomiting POD 0 and 1, They had a shorter hospital stay of 7.7 h; Dexamethasone group demonstrated greater ROM on POD 1 and mobilized a longer distance that the control group; Decrease in total inpatient opioid consumption and need for patient controlled rescue analgesia in the Dexamethasone group. Total morphine consumption in the first 24 h were comparable.No long-term benefits seen with usage of Dexamethasone	No adverse events	Allergic reactions; Bilateral TKA procedures; Chronic opioid usage
Tsukada et al. [35]	Double blinded Randomized Controlled trial	75 patients 38—Methylprednisolone group 37—Saline group	PAI—300 mg Ropivacaine; 8 mg Morphine; 0.3 mg Epinephrine; 50 mg Ketoprofen In the one group they added 40 mg Methylprednisolone (MP) and the second group they only added Normal Saline	MP group had lower cumulative pain scores in the 24 h postoperative. No difference in VAS score at rest between groups but VAS score lower with activity in the first 24 h in the MP group; Some trend of better flexion and extension angle in the MP in the first 10 days	No adverse events. 5 patients develop a peroneal palsy due to the injection at the area—all resolved within 24 h	Poorly controlled DM(HBA1C > 7); Contraindications to spinal anesthetic;Regular opioid usage, Renal insufficiency; Prolonged Q-interval
Christensen et al. [36]	Double blinded prospective Randomized Controlled trial	76 patients MP group 39 patients; No steroid group—37 patients	PAI—80 mg Bupivacaine Hydrochloride; 4 mg Morphine, 0.3 mg Epinephrine; 100ug Clonidine; 750 mg Cefuroxime MP group received an additional 40 mg MP and the no steroid group received Saline	LOS was statistical better in the MP group; No difference between the groups regarding total narcotic consumption on first day or pain score on the first 24 h or upon discharge between the groups No difference in ROM between the two groups	Three patient in the MP had complications compared o none in the no steroid group. The complications included joint sepsis with subsequent death; 2 MUA	Renal disease, Allergic reactions to drugs used, chronic opioid usage, Rheumatoid arthritis; history of a knee infection
Wang et al. [30]	Prospective double blind randomized controlled trial	102 patients: 50 control group and 52 in corticosteroid group	PAI—0.2% Ropivacaine; 2ug/ml Epinephrine Steroid group—0.1 mg/ml Dexamethasone (DM)	VAS scores were statistical lower at 6 + 12 h at rest in the DM group and in the first 24 h with activity. DM had statistical lower overall morphine consumption; IL-6 + CRP was statistical lower in the DM in the first 2 days postoperatively; Longer ambulation and better ROM in the DM only on POD 1.No difference in LOS, nausea + vomiting or glucose levels	No difference in complication rates between the two groups	Other diagnosis than OA (i.e. RA, traumatic arthritis); Allergic reaction; Flexion or varus–valgus deformity > = 30 degrees; history of knee surgery or infection; history of excessive opioid or alcohol consumption; history of narcotic dependency; Psychiatric illness or cognitive impairment; Thrombotic events
Sean et al. [33]	Prospective double blind randomized controlled trial	100 patients; 50 steroid group and 50 patients in the control group	PAI—0.5 ml/kg of 0.5% Bupivacaine with 1:200 000 Epinephrine; Steroid group received 40 mg Triamcinolone acetonide	Significant less pain POD 2—5 in the steroid group; Less morphine consumption in the steroid group; in the steroid group they observed decrease LOS,quicker ability to achieve straight leg raise and better ROM which was significant POD 2 onward. The ROM persisted to be better even at 6 months postop. At 2 years no difference in clinical outcome between the groups	Both groups had 1 infection with a no culture in the steroid group while the control group grew MRSA infection	Diabetes, immunodeficiency, previous surgery to the knee; hypothyroidism, renal failure or allergies to medications
Yue et al. [31]	Prospective randomized controlled study	72 patients; 36 in the steroid group and 36 in the control group	PAI—30 ml of 0.75% Ropivicaine; 0.5 ml of 1:1000 Epinephrine Steroid group—1 ml Betamethasone; The steroid group injected the mixture in thee periarticular tissues but did not inject the steroid in the subcutaneous tissues	No difference in VAs at rest or activity; No difference in ROM or overall morphine consumption; KSS was statistically better at the 1 and 3 month mark in the steroid group but similar after 6 months. Celecoxib usage was shorter in the steroid group	No complications in either group	Know allergies to the medications; Major systemic illnesses (heart failure, renal impairment); psychological problems, narcotic dependence; chronic opioid users
Kwon et al. [26]	Randomized controlled trial	76 staged knee replacements. the first knee was randomized to either receive steroids or no steroids. The second staged knee then received the opposite treatment	PAI—300 mg Ropivicaine; 10 mg Morphine Sulfate; 30 mg Ketorolac, 300ug of 1:1000 Epinephrine Steroid group—40 mg Triamcinolone Acetonide	Statistical lower pain on the night of the operation in the steroid group; Steroid group demonstrated earlier straight leg raise ability, but no difference in maximal flexion; No difference in rescue medication; No difference in flexion,patient satisfaction or PROMS at 6 months	No difference in complication rates	GI bleeding, congestive heart failure, Delirium or failed spinal anesthesia
Kim et al. [32]	Prospective randomized double blind study	270 patients—divided in 6 groups of 45 patients each	All the groups received 400 mg Ropivicaine + 0.6 mg Epinephrine; Group 1—as above; Group 2—Morphine (5 mg) in addition; Group 3 Ketorolac (30 mg) in addition; Group 4 Morphine and Ketorolac in addition; Group5 Morphine, Ketorolac and Methylprednisolone (40 mg) in addition	Lower pain scores first 12 h in Group 4 + 5 compared to the other groups; No difference between Group 4 and 5 however. Lower opioid consumption in the first 24 h in group 4 + 5 but total opioid consumption the same; Group 5 demonstrated lower CRP values until POD 4 and better ROM until POD 2	No difference in complication rates	Inflammatory arthritis, previous surgical procedure to the knee; allergy to the medications

The increase in range of motion with the addition of GCS still needs to be clarified with an equal number of studies demonstrating benefits [28–30, 32, 33] compared to studies showing no apparent benefit [25–27, 31, 36].

Most studies demonstrated a decrease in length of stay in the cohort of patients receiving GCS in their PAI [29, 33, 36] compared to only one study, which did not show any benefit [31].

GCS added to the PAI is a safe option, with most studies not demonstrating an increase in adverse events [26, 28, 30–33, 35]. Two studies did show complications after GCS administration. The first study had one periprosthetic joint infection (PJI) with triamcinolone acetonide [25], and the second study demonstrated one PJI and two manipulations under anesthesia with methylprednisolone [30].

## Ketorolac (Toradol)

We identified 12 articles exploring the effectiveness of ketorolac (Toradol; ketorolac tromethamine) as an element of periarticular injection (PAI) for total knee arthroplasty (TKA) (Table 4) [3, 37–47]. Ketorolac is a non-steroidal anti-inflammatory drug (NSAID) that has been an additive agent to various high-volume local infiltration analgesia cocktails, often including additional medications such as ropivacaine and epinephrine [48]. However, the efficacy of the individual elements of such regimens has limited evidence [37]. Ketorolac is a non-specific cyclooxygenase (COX) inhibitor, limiting the conversion of arachidonic acid to thromboxane, prostacyclin, and prostaglandins, thus decreasing sensitization of afferent nerves [49]. There is also evidence of an impact on the central hypothalamic prostaglandin response by inhibiting prostaglandin synthetase systems [50]. It was first approved for parenteral use in 1990 and thus has been available as an anti-inflammatory for over three decades. Ketorolac has a rapid onset of action following IM and IV administration, with peak analgesic effects at 75–150 min [49]. The half-life of ketorolac is 5–6 h [49]. Adverse effects of ketorolac are similar to those of other NSAIDs, including gastrointestinal bleeding, nausea/vomiting, peptic ulceration, renal failure and increased bleeding due to inhibited platelet function [49], which may limit its effectiveness in many patients receiving total knee arthroplasty.

**Table 4:** Articles included in the review analyzing the use of ketorolac in periarticular infiltrations

Name	Type of study	Number of patients	Groups	Dosage of Ketorolac	Duration of Ketorolac	Findings	Complications
Nikhar et al. [2]	RCT	75 total knee arthroplasty patients	(1) (D) Ropivacaine/Epinephrine + Dexmedetomidine (2) (K) Ropivacaine/Epinephrine + Ketorolac (3) (C) CONTROL –Ropivacaine/Epinephrine	30 mg periarticular infiltration	343.00 min ± 144.45 min pain relief	Ketorolac demonstrated better postoperative pain score, duration of analgesia, and decreased epidural opioid use compared to control and ropivacaine + dexmedetomidine	No complications
Andersen et al. [37]	RCT	60 total knee arthroplasty patients	(1) Intraoperative 150 mL ropivacaine + 30 mg Ketorolac with 8 intra-articular injections postoperatively containing 15 mg Ketorolac q6h (2) Intraoperative 150 mL ropivacaine + 1 mL saline with 8 intra-articular injections postoperatively containing 0.5 mL Saline (CONTROL)	30 mg periarticular infiltration	24–48 h with statistically significant change	Ketorolac demonstrated decreased PCA opioid use and prolonged time to first use of PCA, decreased postoperative pain intensity score while walking and at rest, and decreased time to home readiness in the ropivacaine + ketorolac group vs. control	1 × patient with postoperative hematoma in ketorolac group
Hannon et al. [38]	Meta-analysis	Meta-analysis of 60 studies	Inclusion criteria included English, human studies that included the use of PAI in patients receiving either TKA or THA, containing a control group and providing quantitative outcomes. Meta-analysis included 3 high-quality studies [31, 52, 67] with ketorolac as an additive to PAI in TKA only	30 mg periarticular infiltration	Various	Intraoperative PAI with Ketorolac leads to decrease postoperative pain, but effect on opioid usage is unclear – 2/3 studies found no difference in opioid usage with the addition of ketorolac. The addition of Ketorolac to PAI contain long-acting analgesics provides additional benefit compared to PAI without ketorolac	Various
Apinyankul et al. [39]	RCT	56 patients receiving TKA	1. 60 mg Ketorolac as an additive to unspecified PAI cocktail 2. 80 mg triamcinolone acetonide as an additive to unspecified PAI cocktail	60 mg periarticular infiltration	no significant difference in pain relief in comparison at 0, 24, 48, 72 h postop	Ketorolac showed no significant difference in morphine consumption, VAS scores, postoperative knee extension or straight leg tests immediately after surgery, 24 h, 48, or 72 h postoperatively when compared to the triamcinolone additive. Postoperative knee flexion was decreased in the ketorolac injection group compared to the triamcinolone group	Not documented
Hinzpeter et al. [40]	RCT	48 patients receiving TKA	1. Periarticular infiltration receiving 40 µg of gonyautoxin 2/3 (GTX 2/3) in saline (study group) 2. Periarticular infiltration using 300 mg levobupivacaine, 1 mg epinephrine, and 60 mg Ketorolac	60 mg periarticular infiltration	no significant difference in pain relief in comparison at 0, 6, 12, 36 and 60 h postop	Ketorolac showed no significant difference in morphine consumption although median PCA use in the control group was 9 mg (range 0–54 mg) comparted to 16 mg (range 0–62 mg) to the GTX 2/3 group (p = 0.40). Ketorolac showed improvement of range of motion at 6 and 12 h, no change after 36 h	No difference in complications, side effects or length of stay in hospital
Kopitko et al. [41]	RCT	161 patients receiving TKA	1. Nerve blockade using bupivacaine completed after completion of surgery *n* = 50 2. (LAI) PAI performed during surgery including 10–10 mL 20 mg lidocaine with 0.01 mg adrenaline and 100 mg ropivacaine, 500 mg tranexamic acid, and 30 mg Ketorolac *n* = 52 3. Control group receiving neither PAI or nerve blockade *n* = 59	30 mg periarticular infiltration	significant decrease in pain at 4–8 h, 24–36 h postop	42% of patients receiving PAI had tolerable pain between 4 and 8 h postop, 0% had severe pain based on numerical rating scores (NRS). 3% of PAI patients displayed tolerable pain compared to 19% in the nerve blockade group and 10% in the control group with 0% of patients showing severe NRS scores. PAI groups also showed significantly lower decreases in hemoglobin. The PAI group showed increased use of 100 mg IV Tramadol at 4–8 h and increased use of 50 mg IV Tramadol at 24–36 h, but no significant increases in other medications	No complications
Laoruengthana et al. [42]	RCT	54 patients receiving simultaneous bilateral TKA	1. 1^st^ Knee would receive 50 mg bupivacaine + 30 mg Ketorolac, 2^nd^ Knee another unspecified mixture 2. 1^st^ Knee would receive 50 mg bupivacaine only, 2nd Knee another unspecified mixture	30 mg intraoperative periarticular infiltration plus 30 mg IV q8h for the first 48 h	Decreased pain for 96 h postoperatively	Ketorolac group showed statistically improved postoperative VAS pain scores from 12 to 96 h. Ketorolac group showed higher degrees of knee flexion and straight leg raise. 61.11% of patients favored the knee receiving the Ketorolac compared to the contralateral knee	No complications
Laoruengthana et al. [43]	RCT	100 patients receiving TKAs	1. Patients received PAI containing 100 mg Bupivacaine and 30 mg Ketorolac and postoperative IV Ketorolac (*n* = 50) 2. Patients received PAI containing 100 mg of bupivacaine with 20 mg of parecoxib and postoperative IV parecoxib (*n* = 50)	30 mg intraoperative periarticular infiltration plus 30 mg IV q12h for the first 48 h	pain was decreased in the ketorolac group 6 h postop	Ketorolac group showed significantly decreased VAS scores comparted to the parecoxib group at 6 h postoperative. Levels of total morphine consumption at 24–48 h were comparable. Total perioperative blood loss and hemoglobin change was increased in the ketorolac group but showed no difference in blood transfusion	1 × DVT in Ketorolac group, 1 × PJI in parecoxib group. No statistical difference in complications between groups
Liu et al. [44]	RCT	134 patients receiving TKAs	1. (A) patients receiving PAI cocktail containing 400 mg ropivacaine, 30 mg ketorolac, 0.3 mg adrenaline, 5 mg morphine, diluted in normal saline 2. (B) patients receiving PAI with 200 mg bupivacaine, 40 mg methylprednisone, 0.3 mg adrenaline, 5 mg morphine, diluted in normal saline	30 mg periarticular infiltration	Group A displayed lower VAS scores improved over 72 h and improved range of motion over 14 days	Group A (PAI containing ketorolac) showed significantly decreased VAS scores at 6 h, 24 h, 48 h, and 72 h compared to group B. Group A also displayed improved joint range of motion on the 3rd, 7th, 10th, and 14th days	No complications
Danoff et al. [45]	RCT	26 patients receiving simultaneous bilaterally TKA	Patients receiving bilateral TKA with one knee receiving 266 mg liposomal bupivacaine 30 mL of 0.25% and bupivacaine (EXP), and the contralateral knee receiving 250 mg ropivacaine, 0.5 mg epinephrine, 30 mg ketorolac, and 0.08 mg clonidine (ROP)	30 mg periarticular infiltration	No statistical difference during the first two days postoperatively	There was no difference in visual analog scales or functional recovery between groups	No complications
Tammachote et al. [47]	RCT	64 patients receiving TKA	1. (M) Group receiving multimodal PAI containing 150 mg levobupivacaine, 30 mg ketorolac, and 5 mg morphine 2. (S) Group receiving PAI with 150 mg levobupivacaine only	30 mg periarticular infiltration	(M) showed decreased pain level in first 4 h and less morphine consumed in 8 h. (M) demonstrated 254 ± 155 min pain relief vs 148 ± 82 min pain relief in (S) group	Significantly decrease VAS score in group containing levobupivacaine, ketorolac, and morphine (M) group compared to single analgesic (S). (M) also showed decrease morphine use in first 8 h, and approximately 2 h longer until the first request of analgesia	No complications
Motififard et al. [46]	RCT	110 patients receiving TKA	1. Patients receiving PAI cocktail containing 50 mg bupivacaine hydrochloride, 1 mL morphine sulfate 10 mg/mL, 3 mcg epinephrine (1:1000), and 30 mg ketorolac. (*n* = 57) 2. Patients receiving PAI containing 300 mcg epinephrine (1:1000) only (*n* = 53)	30 mg periarticular infiltration	VAS scores obtained at 24 h, 48 h, and 6 weeks postop	Bupivacaine + morphine + epinephrine + Ketorolac group showed increase Knee Society Score after 6 weeks. There was significantly lower VAS scores and significantly higher ROM in the PAI cocktail group compared to the epinephrine control at 24 h, 48 h and six weeks	No complications related to intervention

Ketorolac's recommended IM and IV dose is 30 mg as a one-time dose or 30 mg every 6 h up to a maximum total of 120 mg in 24 h [51]. Still, some studies also describe 60 mg as a dosage option[39, 40] with a possible dose-dependent response [49, 52]. Many studies included visual analog scale (VAS) scores and opioid usage as measures of effectiveness for postoperative pain management. All but two studies [39, 45] that explored subjective pain scores found improved postoperative pain when ketorolac was added to the PAI. Decreased opioid usage was reported by Nikhar [3], Tammachote [47] and Andersen [37], but opioid usage was otherwise not significantly reduced [38–40]. Improvements in postoperative function, including range of motion [39, 40, 42, 44–46] and time to home readiness [37], were also investigated and were generally favorable, with some exceptions [39, 45]. Complications in the studies with ketorolac infiltration were rare, with one episode of hematoma [37] and one episode of DVT [43] highlighted across the studies. There were no documented statistical increases in complication rates for PAIs containing ketorolac. In the studies examined, the duration of action of ketorolac was reliably improved in the first 4–8 h [3, 37, 41–44, 46, 47], and pain relief was seen up to 96 h [43].

Overall, 9 out of the 12 studies did show a benefit of using ketorolac as part of multimodal pain management compared to a management plan not containing ketorolac. Exclusion criteria included previous surgery on the joint in question [37, 40, 42, 43, 46], previous infection [3, 41–43], bilateral knee joint involvement [44, 45], cardiac disease [3, 42, 43, 47], history of venous thromboembolism [42, 43], coagulopathies [3, 37, 40, 44], hepatic [3, 40, 46, 47] or kidney disease [3, 40, 42, 43, 46, 47], gastrointestinal ulcer [42, 43] or hemorrhage [42, 43], cerebrovascular disease [40, 42–44], neurocognitive disorders [41, 44, 47], rheumatoid arthritis [37, 40, 46], or allergies to the medications [3, 37, 40–44, 46, 47]. Patients under these exclusion criteria should be given extended clinical consideration before Toradol is included in periarticular infiltration during TKA.

## Liposomal bupivacaine

Local anesthetics have proven beneficial in intraoperative and postoperative pain relief. Using local drugs, pain relief is mediated by impairing the voltage-gated sodium channels, which leads to decreased depolarization and therefore decreased conduction of pain signals [53]. However, their use is limited by their relatively short duration of action. Bupivacaine is a hepatically metabolized amide-type local anesthetic with an expected analgesic effect of up to 8–10 h [54].

Formulations of bupivacaine encapsulated in liposomes (liposomal bupivacaine) have been developed with the proposed benefit of sustained analgesia. These liposomes are composed of a lipid bilayer which encapsulates the anesthetic. This results in a delayed release of drugs based on lipid permeability and lipid bilayer breakdown prolonging the duration of action [55]. Initial studies have suggested an improvement of up to 72–96 h of effect [55]. The first formulations gained FDA approval in 2011. Subsequently, they began to be studied for the possibility of opioid-sparing effects [55].

Although some studies have shown benefits to liposomal bupivacaine over standard formulations[56], many have failed to show statistically significant improvements in opioid usage, time to discharge, or functional status in long-term follow-up [57–62] (Table 5). Several studies demonstrated no difference in pain experienced with rest [57–63]. Three studies did demonstrate decreased pain with physiotherapy [60, 64, 65], while only one study demonstrated the opposite [66]. Most studies showed no difference in the total narcotic usage [57–61, 64–66]. There were similar satisfaction rates in three studies [57, 62, 63], while only one study demonstrated better satisfaction with LB [56]. This has raised the question of the role of liposomal bupivacaine, especially given the greater-than-average cost of the medication^60^.

**Table 5:** Articles included in the review analyzing the use of liposomal bupivacaine in periarticular infiltrations

Name	Type of Study	Number of patients	Groups and findings	Dosage used	Findings	Complications
Marino et al. [57]	RCT	65 total knee arthroplasty	1. CFNB + PAI of bupivicaine 2. PAI bupivicaine + liposomal bupivicaine	1. continuous femoral nerve block 20 mL 0.5% bolus bupivacaine with continuous infusion -.2% at 8 ml/h for 48 h, spinal anesthetic with 12.5 mg bupivacaine 0.75%, periarticular injection of 60 cc solution of 30 mL 0.5% bupivacaine HCl, 0.5 ml epi 1:200,000 2. PAI 30 ml 0.5% bupivacaine 10 mL NaCl, 0.5 ml epi 1:200,000, 20 ml 266 mg liposomal bupivacaine	No difference in pain at rest, increased pain with maximum flexion in LB vs CFNB at 24 h. Similar patient satisfaction, similar narcotic use overall except CFNB started use of PCA earlier (205.8 min vs 116.5 min). 9.4% vs 57.6% required use of knee immobilizer for knee buckling postop until POD2. Same active knee ROM at 24 h, CFNB better at 48 h (74.1 degree vs 62.74 degree)	1 patient exceeded the toxicity threshold for bupivacaine in CFNB, none in LB. No participants with symptoms of local anesthetic systemic toxicity
Zlotnicki et al. [64]	RCT	80 total knee arthroplasty patients	1. PAI bupiv 2. PAI liposomal bupivacaine 3. Retrospectively reviewed control with no PAI	1. 20 ml 0.5% plain bupivacaine + 70 mL NS 2. 20 mL 0.5% liposomal bupivacaine + 70 mL NS	Decreased pain with PT with LB compared to bupivacaine at 24 h but not 48 h. No difference in total pain medications, ROM on POD1 or at discharge Both superior to no PAI	None reported
Dizdarevic et al. [58]	RCT	25 (interim analysis, 90 total) total knee arthroplasty	1. ACB + PAI bupivacaine 2. ACB + PAI bupivicaine/liposomal bupivicaine	1. Adductor canal block + 40 mL 0.25% bupivacaine PAI 2. Adductor canal block + 40 mL 0.65% exparel, 0.25% bupivacaine	No change in 48 h opioid use, no change in function (measured via 6-Clicks score), no difference for numerical pain rating scale at 24 or 48 h	None reported
Dysart et al. [56]	RCT	139 total knee arthroplasty	1. LIA with LB + bupivacaine 2. LIA with bupivicaine	1. LIA with LB 266 mg/20 mL mixed with bupivacaine 0.5% 20 mL 2. bupivacaine alone	LB group less likely to use opioid rescue medications within first 24 h, reduced opioid consumption over first 24 h (3.5 vs 38.5), reduced pain intensity. More likely to be discharge ready within 12 h of surgery (42.9 vs 27.5%), higher satisfaction with pain treatment. Timed up and go test no difference	No serious adverse events, nausea/dizziness/vomiting similar between groups
Hyland et al. [59]	RCT	59	1. ACB + PAI (ropivicaine, morphine, ketoralac, methylprednisolone) 2. ACB + PAI liposomal bupivicaine	1. PAI: 20 ml 0.2% ropivacaine, 10 mg morphine, 30 mg ketorolac, 40 mg methylprednisolone 2. 60 ml liposomal bupivicaine 266 mg	No difference in number of PT sessions necessary for discharge, total opioid consumption, average pain scores. Average total drug charges significantly higher for liposomal bupivacaine	None listed
Smith et al. [60]	RCT	200 total knee arthroplasty	1. intra-articular slow infusion delivery system (ON-Q) of bupivacaine 2. 20 ml 266 mg liposomal bupivicaine with 40 ml .9% saline + intra-articular slow infusion delivery system (ON-Q) of saline	20 ml 266 mg liposomal bupivicaine with 40 ml .9% saline	No difference in morphine equivalents consumed, hospital stay length, satisfaction pain scores, scores for ability to fall asleep. Bupivicaine infusion had less pain with PT	3 patients with LB required MUA for stiffness, 5 patients for bupivicaine infusion required MUA. 1 LB had wound dehiscence and suspected infection,
Perets et al. [61]	RCT	107 total hip arthroplasty patients	1. PAI with LB + bupivicaine HCl 2. Control of bupivicaine HCl and epinephrine	1. 60 ml 0.25% bupivicaine with epinephrine 2. 20 ml liposomal bupivicaine, 40 ml 0.25% bupivicaine with epinephrine	No statistical significant difference in morphine equivalent use (total or at any time point), no falls for either, no difference in mean time to ambulation, no difference in VAS pain scores	Similar rates of constipation, nausea, vomiting
Johnson et al. [65]	RCT	159 total hip arthroplasty	1. Peripheral nerve block 2. PAI with ropivacaine, ketorolac, epinephrine 3. PAI with liposomal bupivacaine,ketorolac, epinephrine	1. Peripheral nerve block (0.5% bupivicaine with epi 30 mL with bupivicaine infusion 0.2% in PACU 2. PAI with ropivacaine, ketorolac, epinephrine, weight based Ropivacaine of 200–400 mg, 100–300 µg epinephrine, 30 mg ketorolac diluted to 120 ml solution 3. PAI with liposomal bupvicaine, ketorolac, epinephrine, 266 mg liposomal bupivicaine, 30 mg ketorolac, 125 mg bupivicaine, 125 µg epinephrine. diluted to 120 ml	No difference in postoperative pain scores on POD1 morning, LB had lower pain scores than no LB but not lower than PNB. No difference in postoperative opioid use or need for IV analgesic. No difference in hospital length of stay, adverse events during hospitalization. No difference in change of physical/mental composites scores for SF36 over 3 months or pain at rest/with movement	2 falls after discharge in bupivicaine group, 1 fall in liposomal bupivicaine group
Talmo et al. [66]	RCT	373 knee arthroplasty patients	1. Femoral nerve block 2. PAI with liposomal bupivicaine + placebo saline FNB	1. 30 ml 0.25% bupivicaine + 30 ml 0.25% bupivacaine 2. 30 mL 0.25% bupivacaine + 20 ml (266 mg liposomal bupivicaine) + 40 ml saline	Control group had lower pain scores, higher range of motion at 12 h. Liposomal bupivacaine group was more likely to be able to perform straight leg raise at 12 h postoperatively and scored higher in physical function short form 12 score at 3 months post op. No significant difference in median walking distance at 12, 24, 36, 48 h postoperatively. All variables had similar variables at 1 year postoperatively. No difference in pain medication use between the two groups	2 patients in the nerve block group required additional rescue FNB for severe postoperative pain. No difference in complication rates
Alijanipour et al. [62]	RCT	162 total knee arthroplasty patients	1. Liposomal bupivacaine 2. Free bupivicaine	1. 20 ml (266 mg) liposomal bupivicaine with 40 ml NS and 0.5 ml epinephrine 1 mg/ml 2. 20 ml (50 mg) free bupivicaine 0.25% with epinephrine 1:200,000 with 40 ml NS	No difference in postoperative pain scores, narcotic side effects, surgical/medical complications, length of stay, patient satisfaction, or Knee Society Score	Similar complication rates between liposomal bupivicaine and bupivicaine
Ali et al. [63]	Dual center RCT	108 shoulder arthroplasty patients	1. Local liposomal bupivacaine 2. Interscalene nerve block	1. 20 ml suspension of 266 mg LLB with 20 mL saline solution, injected into skin/subcut, deltoid, pectoralis muscle, pericapsular, periarticular + 30 mL 0.5% bupivicaine—epinephrine (given slower onset of action 3. 0.5% ropivacaine for INB, dosed by weight	VAS pain score in INB were better than LLB at 6 h, no difference after 6 h Similar intra-op opioid requirements, INB had fewer morphine milligram equivalents 18 + -12 compared to LLB 36 +—48 over first 24 h, similar results over days 2–4, PACU stay shorter for INB 102 +—53 versus LLB 139 +—77 min No difference i duration of hospital stay or satisfaction with pan control in hospital or at home	No adverse events experienced

The most commonly used dosage in the RCTs examined was 20 mL (266 mg) of liposomal bupivacaine mixed with 40 mL of normal saline solution. Additional variations to the periarticular cocktail include adding epinephrine, ketorolac, or standard bupivacaine solutions.

The most commonly used exclusion criteria in the tabled studies include allergies to amide anesthetics [57, 59, 62, 63, 65, 66], chronic pain or opioid dependence [57, 59, 62, 64–66], abnormal hepatic, renal or cardiac function [56, 57, 65, 66] and elevated BMI [56, 62, 65, 66].

No specific complications were associated with liposomal bupivacaine described in the reviewed studies. Most reported complications involved arthroplasty complications such as infection, wound dehiscence or periprosthetic fracture following a fall [67]. One study involved a patient exceeding bupivacaine's toxicity threshold in a continuous femoral nerve block. However, this patient had no symptoms of systemic local toxicity [57]. The most reported serious complications of bupivacaine are related to cardiac and neurologic toxicity. These are more likely to occur when exceeding the recommended safe dose (2–2.5 mg/kg) but have been described in individuals even at lower doses [53]. The potentially life-threatening side effects highlight the importance of coordination between care team members to avoid reaching toxic doses.

## Morphine

Morphine is an opioid medication that primarily acts through μ opioid receptors [68]. Activation of μ opioid receptors in the central nervous system has a long-standing history of use in pain control and sedation. Opioid receptors in the peripheral nervous system have also been described. However, the mechanism and effects are less well known.

The evidence described in arthroscopy literature shows the benefit of pain control with intra-articular injections. In animal studies, there is evidence for the benefit of local infiltration of morphine [69]. However, our literature review shows limited data for this in human studies (Table 6). Previous systematic reviews on periarticular morphine have identified a scarcity in the number of studies examining peri/intra-articular morphine [70]. An equivalent number of studies examined in Table 6 showed benefit [32, 47, 71–73] and no difference [74–78] in pain scores. The duration of pain improvement varied between 2 and 24 h [32, 47, 71–73]. Most studies showed a better range of motion with Morphine usage [47, 74–78]. This was similar to opioid consumption postoperatively. Six studies demonstrated less morphine consumption [32, 47, 71–73, 75], while some demonstrated no difference [74, 76–78]. This reduction in opioid consumption was observed in the first 48 h. Dosages used in studies reviewed showed ranges from 1 mg of morphine up to 10 mg. Most studies used 5 mg Morphine Sulfate [32, 47, 74, 78]. Three studies used a weight-based regime of 0.1 mg/kg [75–77]. Heine et al.’s [72] results suggested a likely dose-dependent effect in the case of intra-articular morphine.

**Table 6:** Articles included in the review analyzing the use of morphine in periarticular infiltrations

Name	Type of study	Number of patients	Groups and Findings	Dosage used	Findings	Complications
Han et al. [74]	RCT	90 TKA	1. Spinal anesthetic + synovial injection of Ropivacaine/epinephrine/morphine 2. Spinal anesthetic + synovial injection of Ropivacaine/epinephrine 3. Spinal anesthetic + synovial injection of saline	1. 40 ml 300 mg ropivacaine with 0.25 mL of 1:200,000 epi, 0.5 mL of 5 mg morphine, 9.25 mL 0.9% NS 2. 40 ml 300 mg ropivacaine with 0.25 mL of 1:200,000 epi, 9.75 mL 0.9% NS 3. 50 mL of 0.9% NS	In the context of epidural PCA, no difference in VAS pain scores at rest or exercise. No difference in rescue tramadol use for pain, no difference in booster PCA use before epidural catheter removal. No difference in range of motion observed	No statistical difference in nausea/vomiting. No other complications reported
Garcia et al. [71]	RCT	50 TKA	1. Intra-articular morphine injection 2. Intra-articular saline injection	1. 1 ml (10 mg) morphine in 19 mL NS 2. 20 mL NS	Intra-articular morphine had lower pain scores reported at 2 and 6 h in treatment group, but otherwise had similar pain scores. Treatment group also had decreased mean 24-h morphine consumption (12.2 mg vs 20.6 mg), decreased time to request of first rescue medication	No statistical difference in incidence of nausea, vomiting, somnolence
Kim et al. [32]	RCT	256 TKA	All patients received spinal anesthetic + PAI of 1. Saline, epinephrine, cefazolin 2. Ropivacaine 3. Ropivacaine, morphine 4. Ropivacaine, ketorolac 5. Ropivacaine, morphine, ketorolac 6. Ropivacaine, morphine, ketorolac, methylprednisolone	1. 1 g cefazolin (10 mL), and 0.6 mg epinephrine (1:1000, 0.6 mL), 49.4 mL NS 2. Ropivacaine 180 mg (24 mL) + 36 mL NS 3. Ropivacaine 180 mg (24 mL) + 5 mg (5 mL) morphine sulfate + 31 mL NS 4. Ropivacaine 180 mg (24 mL) + ketorolac 30 mg (1 mL) + 35 mL NS 5. Ropivacaine 180 mg (24 mL) + 5 mg (5 mL) morphine sulfate + ketorolac 30 mg (1 mL) 30 mL NS 6. Ropivacaine 180 mg (24 mL) + 5 mg (5 mL) morphine sulfate + ketorolac 30 mg (1 mL) + methylprednisolone 40 mg (1 mL) + 29 mL NS	All groups had lower pain scores compared to control for first 6 h after surgery. Combinations of Ropivacaine/ketorolac/morphine ± methylprednisolone showed improvement in pain scores for 12 h as well as lower opioid consumption over the first 24 h post-surgery. No statistical difference in total opioid consumption over the hospital stay between any group	No difference in nausea/vomiting/urinary retention/wound problems/deep infection
Heine et al. [72]	RCT	31 arthroscopic	1. IA (intra-articular bupivacaine 2. IA bupivacaine and morphine 3. IA bupivacaine and higher dose morphine	1. IA 100 mg bupivacaine 2. IA 100 mg bupivacaine, 1 mg morphine 3. IA 100 mg bupivacaine, 3 mg morphine	Bupivacaine + 3 mg morphine had improved pain at rest at 12 h and day 1, and improved at 12 h/day 1/day 2 on standing, improved walking scores. Both morphine groups had received less analgesia then bupivacaine alone	Report increased nausea and somnolence with addition of morphine, no statistical analysis reported
Li et al. [73]	RCT	120 TKA	1. Cocktail with morphine + epidural morphine 2. Cocktail with morphine 3. Cocktail without morphine	1. 100 mg ropivacaine, 6 mg dipropionate betamethasone, 15 mg betamethasone sodium phosphate, 8 mg morphine, saline to 60 ml + 2 mg epidural morphine 2. 100 mg ropivacaine, 6 mg dipropionate betamethasone, 15 mg betamethasone sodium phosphate, 8 mg morphine, saline to 60 ml 3. 100 mg ropivacaine, 6 mg dipropionate betamethasone, 15 mg betamethasone sodium phosphate, saline to 60 ml	Addition of morphine to cocktail had lower pain scores than cocktail without morphine although worse than morphine cocktail with epidural. Lower pain at rest, lower tramadol requirements. All groups had similar quadricep strength	No statistical significance in nausea, vomiting or metoclopramide usage
Wang et al. [75]	RCT	100 TKA	1. PAI of ropivacaine, epinephrine 2. PAI of ropivacaine, epinephrine, morphine	1. 0.2% ropivacaine and 2.0 μg/mL epinephrine 2. 2% ropivacaine, 2.0 μg/mL epinephrine, and 0.1 mg/mL morphine hydrochloride	Lower morphine use in first 24 h and total. No difference in VAS pain scores, knee ROM, quadricep strength, or daily ambulation distance	1 patient in morphine group developed 1 day of foot drop
Iwakiri et al. [76]	RCT	53 undergoing bilateral TKA (106 TKA total)	1. PAI with Ropivacaine/epinephrine/ketoprofen/methylprednisolone 2. Ropivacaine/epinephrine/ketoprofen/methylprednisolone/morphine	1. 0.5 mg/mL of ropivacaine [20 mL], 1:1000 epinephrine [adrenaline; 0.1 mL], 25 mg of ketoprofen [1.25 mL], 20 mg of methylprednisolone sodium [0.3 mL], and 8.35 mL of normal saline solution 2. 0.5 mg/mL of ropivacaine [20 mL], 1:1000 epinephrine [adrenaline; 0.1 mL], 25 mg of ketoprofen [1.25 mL], 20 mg of methylprednisolone sodium [0.3 mL], and 8.35 mL of normal saline solution, 0.1 mg/kg morphine	No difference in pain VAS scores at rest or in motion, no difference in ROM, thigh swelling	Comparison was made between TKA of a single patient so nausea/vomiting reported as aggregate. No surgical site infection, nerve palsy, DVT
Iwakiri et al. [77]	RCT	102 TKA	1. Ropivacaine/epinephrine/ketoprofen/methylprednisolone 2. Ropivacaine/epinephrine/ketoprofen/methylprednisolone/morphine	1. .5 mg/mL of ropivacaine [40 mL], 1:1000 epinephrine [0.1 mL], 50 mg of ketoprofen [2.5 mL], 40 mg of methylprednisolone sodium [0.6 mL], and 16.8 mL of normal saline solution 2. 0.5 mg/mL of ropivacaine [40 mL], 1:1000 epinephrine [0.1 mL], 50 mg of ketoprofen [2.5 mL], 40 mg of methylprednisolone sodium [0.6 mL], 10 mg morphine [1 ml] and 15.8 mL of normal saline solution	No difference in VAS pain scores at rest or in motion, use of rescue analgesia, ROM, thigh swelling	Morphine group had statistically significant increased nausea between 30 min to 9 h and significantly more vomiting episodes and anti-emetic usage
Tammachote et al. [47]	RCT	64 TKA	1. Levobupivacaine/ketorolac/morphine/epinephrine 2. Levobupivacaine only	1. levobupivacaine 150 mg, ketorolac 30 mg, morphine 5 mg, 0.6 mg epinephrine 2. Levobupivacaine 150 mg	Morphine group had decreased pain scores at rest over the first 4 h after surgery, less morphine consumption over the first 48 h with larger effect in the first 8 h. ROM, length of stay and re-admission were similar	No wound complications in either group. No other complications reported
Mauerhan et al. [78]	RCT	105 TKA	1. Saline 2. Morphine 3. Bupivacaïne 4. Morphine/Bupivacaine	1. 10 mL saline 2. 5 mg morphine sulfate 3. 50 mg bupivacaine 4. 5 mg morphine sulfate/50 mg bupivacaine	All intra-articular injections led to improved pain scores compared to placebo but no difference in pain scores between test groups. No difference in PCA usage. Analysis of combined morphine/bupivacaine groups shows some difference in pain scores only at 4/6 h group	No complications reported

Given the mixed data, its use should be considered in the context of possible adverse effects of systemic morphine, including nausea, vomiting, sedation, constipation, and pruritus being most described. Iwakiri et al.’s [77] study showed concerns about the possibility of nausea, vomiting and increased requirements for anti-emetics even in the case of local infiltration. However, there is again heterogeneity in the data as other articles reviewed showed no statistical difference in the development of nausea/vomiting postoperatively [32, 47, 71–76, 78].

The exclusion criteria most commonly utilized include but are not limited to morphine sensitivity [32, 47, 73–78]; alcohol or narcotic dependency [72–77]; previous surgical procedure involving the same knee [32, 75–77]; inability to do a spinal anesthetic [47, 72, 73, 78]; and a history of cardiac or thrombotic events [47, 75–77].

## Ropivacaine

Ropivacaine is a local anesthetic agent initially described in reports of the first periarticular cocktails used in joint arthroplasty. Subsequent studies have used different drug cocktails and combinations of local anesthetics and compared their efficacy to ropivacaine [44, 45, 79, 80]. Ropivacaine is commonly used in epidurals and major nerve blocks. It is a long-acting local anesthetic that reversibly inhibits sodium ion influx in nerve fibers [81]. Some properties of ropivacaine that make it unique are that it is less lipophilic than other local anesthetics and, therefore, less likely to penetrate large, myelinated motor fibers, selectively acting on the nociceptive fibers [81]. It usually has an onset of action in 1–15 min with a duration of 2–6 h[82]. It is reported to have significantly less cardiotoxicity and neurotoxicity [81, 83, 84]. It is generally well tolerated with few side effects [85]. The most commonly reported adverse reactions are hypotension, nausea and vomiting [85].

When used in periarticular blocks in the context of arthroplasty, ropivacaine has demonstrated the capacity to lower patient-reported pain scores in a dose-dependent manner [82]. Newer literature has sought to compare its efficacy to bupivacaine and liposomal bupivacaine (Table 7). Based on our literature review, there has been no consistent statistically significant difference in the performance of these three drugs (see liposomal bupivacaine section) [45, 67, 79, 80]. One of the highlighted studies did show better pain management with ropivacaine compared to bupivacaine [44].

**Table 7:** Articles included in the review analyzing the use of ropivacaine in periarticular infiltrations

Name	Type of study	Number of patients	Groups and findings	Administration	Findings	Complications
Danoff et al. [45]	RCT	29 bilateral TKA patients, 58 knees	Group 1: Liposomal bupivacaine and bupivacaine (EXP) Group 2: Ropivacaine, epinephrine, ketorolac and clonidine	Periarticular infiltration	No significant difference in visual analog scale pain scores at any time point, no difference in functional recovery on POD 0–2	No complications
Amundsonet al. [67]	RCT	165 patients	Group 1: femoral catheter and sciatic nerve block Group 2: Ropivacaine based periarticular injection Group 3: Liposomal bupivacaine based periarticular injection	Group 1: Bupivacaine 0.5% bolus preop, 0.2% bolus in PACU. 0.25% single injection to sciatic nerve Group 2: Ropivacaine 200–400 mg (weight based), 100 mcg epinephrine, 30 mg ketorolac Group 3: 266 mg liposomal bupivacaine, 30 mg ketorolac, 125 mg bupivacaine, 125 mcg epinephrine	Ropivacaine and liposomal bupivacaine based periarticular injection provide comparable pain control on POD 1 and 2 to femoral catheter and single injection sciatic nerve block	No complications
Liu et al. [44]	RCT	134 patients undergoing UKA	Group 1: Ropivacaine, ketorolac, adrenaline, morphine, NS Group 2: Bupivacaine, methylprednisolone, adrenaline, morphine, NS		Group one had significantly lower VAS scores at 6, 24, 48 and 72 h. ROM up to 14 days was superior	No complications
Leeuw et al. [79]	RCT	37	Group 1: 0.5% bupivacaine with 1:200,000 epinephrine, group 2: 0.5% ropivacaine	Posterior lumbar plexus block	Not clinically significant lower pain scores in ropivacaine group at 8, 12 and 24 h	No complications
Hungerford et al. [80]	RCT	100 patients; 54 in control and 46 in experimental	Group 1: Liposomal bupivacaine mixed with bupivacaine Group 2: Ropivacaine (control drug)	Adductor canal block	No statistically significant difference in pain scores at 24, 48 and 72 h or length of stay	No complications
Xiao et al. [86]	RCT	120 THA patients	3 groups (1) LIA in deep and superficial fascia (2) LIA in all layers (3) Control	80 mL 0.25% ropivacaine Group 1: 40 mL after suturing deep fascia, 40 mL after suturing superficial fascia Group 2: 40 mL to deep tissues, 40 mL to superficial tissues (including skin and subcutaneous) Group 3: No infiltration	LIA with ropivacaine groups has lower resting VAS scores than control group at 2 and 6 h and lower VAS scores when mobilizing at 6 and 12 h. LIA groups have higher patient-reported satisfaction scores. Opioid consumption was similar in all 3 groups	No complications
van Haagen et al. [80]	RCT	128 TKA patients	Group A: 300-mg ropivacaine/600–300–300-mg gabapentin. Group B: 150-mg ropivacaine/600–300–300-mg gabapentin. Group C: 300-mg ropivacaine/300–100–100-mg gabapentin. Group D: 150-mg ropivacaine/300–100–100-mg gabapentin	Ropivacaine administered as LIA. Gabapentin administered po on first day after surgery	Significant difference in pain scores between group A and B, suggesting altering dose of ropivacaine influences the course of pain and gabapentin does not	None
Teratani [84]	RCT	128 arthroscopic rotator cuff repair patients	Cocktail group: 0.75% ropivacaine, 5 mg morphine, 0.3 mg epinephrine, 2 mg betamethasone, NS to a total of 42 mL. Control group: 0.75% ropivacaine and NS to a total of 42 mL	Injection into glenohumeral joint, subacromial bursa, suprascapular nerve, and deltoid muscle	VAS pain scores were lower in the cocktail group at 8, 16 and 24 h. No apparent detrimental effects on tendon healing	Lower suppository use in cocktail group, higher nausea rate in control group but not statistically significant

The usual dosage for ropivacaine used in a field block with 0.5% concentration is 5–200 mg (1–40 ml) [82]. In the tabled studies, the dosing of ropivacaine was weight-based, with Ropivacaine concentrations varying between 0.25 and 0.75% [86]. Van Haagen et al. [82] specifically looked at the effects of varying concentrations of ropivacaine in the periarticular block, comparing doses of 150–300 mg, and suggested that the higher dosage provided better pain relief.

Although ropivacaine use is commonly associated with nausea and vomiting, only one study commented on this side effect. Teratani et al. [87] reported that the group that received only ropivacaine in normal saline instead of a cocktail (including morphine, epinephrine, and betamethasone) said higher rates of nausea and vomiting postoperatively. However, this did not achieve statistical significance.

Ropivacaine is metabolized in the liver, and the metabolites are excreted through the renal system [82]. Therefore, dose adjustments should be considered for patients with hepatic or renal involvement. Caution should also be practiced in elderly debilitated patients and patients with cardiac disease [82].

## Tranexamic acid (TXA)

Using the search criteria, there were five randomized control trials [88–92] evaluating the effect of TXA as an additive to periarticular infiltration (Table 8). Tranexamic acid (TXA) is a competitive inhibitor of plasminogen, a component of the fibrinolytic pathway necessary for hemostasis [91]. As such, TXA has shown promise in orthopedic surgery in reducing bleeding-related complications, such as hemarthrosis or postoperative bleeding. In most cases, TXA is administered intravenously, but TXA can also be administered intra-articularly [93–95], using drain clamping [92, 96–99], or via intraoperative TXA soak [91].

**Table 8:** Articles included in the review analyzing the use of tranexamic acid in periarticular infiltrations

Name	Type of study	Number of patients	Groups	Dosage of Tranexamic Acid	Duration of Tranexamic Acid	Findings	Complications
Peng et al. [91]	RCT	93	1. IV administration of 1 g TXA (*n* = 47) 2. Periarticular administration of 1 g TXA (*n* = 46)	1 g	Complications were monitored day 3 and day 14	TXA administration via PAI significantly decreased total blood loss and hidden blood loss compared to IV TXA. There was no difference in drainage volume	No venous thromboembolism. No severe complications reported
Hishimura et al. [92]	RCT	109	1. (L) Local injection of TXA (*n* = 57) 2. (D) Drain Clamping (*n* = 52)	10 mg/kg	Assessment period 14 days, VTE evaluated at 7 days, Hgb levels evaluated at postoperative day 3	Total blood loss showed non-inferiority in the PAI group vs. drainage clamping, however, TXA was superior for suppressing bleeding	Not reported
Kim et al. [88]	RCT	240	1. Intravenous TXA (*n* = 80) 2. TXA administered in a periarticular injection (*n* = 80) 3. Combination of Intravenous and periarticular injection of TXA (*n* = 80)	1 g	Evaluation period 1–5 days postoperatively	Intravenous and periarticular administration of TXA showed comparable effects. Combination of IV and periarticular TXA showed added effect	No differences in complications between groups. No differences in needs for transfusion
Zhang et al. [90]	RCT	218	1. Intra-articular injection of TXA, periarticular injection of placebo (*n* = 52) 2. Intra-articular injection of TXA and periarticular injection of TXA (*n* = 53) 3. Intra-articular injection of placebo, periarticular injection of TXA (*n* = 50) 4. Intra-articular injection of placebo and periarticular injection of placebo (*n* = 55)	1 g	Range of motion, and hemoglobin explored on postoperative day 1. VAS assessed on postoperative day 2. DVT assessed 7 days postoperative	Combined use of periarticular and intra-articular injection of TXA significantly reduced total blood loss. Combined method also shows improved visual analogue scales and range of motion in the early postoperative period (48 h), but not in the late postoperative periods (6 months)	No thromboembolic events. No differences in wound-related complications between groups
Pinsornsak et al. [89]	RCT	108	1. Patients receiving 15 mg/kg Periarticular infiltration of TXA (*n* = 36) 2. Patients receiving 2 g intra-articular TXA (*n* = 36) 3. Patients receiving no TXA (*n* = 36)	15 mg/kg	No significant difference in total blood loss between periarticular and intra-articular groups at 48 h. Serum TXA elevated at 2 h and 24 h postoperative	Total blood loss, hemoglobin decreases, and blood transfusion rates were decreased in the periarticular and intra-articular groups compared to control. Serum TXA levels were significantly higher in the intraarticular group vs. the periarticular group	No differences in complications of periarticular and intra-articular groups. 14% of control group showed subcutaneous ecchymoses No venous thromboembolism

Peng et al. [91] hypothesized that TXA might be better functionally used as an element of PAI to infiltrate damaged tissues locally, prolonging the drug’s effects. They proposed that local infiltration may also reduce the risk of adverse effects [91], which include nausea, intraoperative hypotension, deep venous thrombosis, and blood transfusion [88–90, 92]. Peng et al. showed a significant decrease in HBL and blood loss in the PAI TXA group compared to the TXA administered intravenously [91]. Kim et al. showed decreased bleeding when PAI and IV administration are combined [88]. Pinsornak et al. [89] also showed decreases in blood loss and transfusion rates compared to intra-articular TXA.

However, the effect of TXA administered in a PAI may be limited to reduced blood loss. All the studies included showed decreased [89–91] or equivalent bleeding [88, 92] compared to other routes of TXA administration. Only one study by Zhang et al. [90] showed improved VAS scores and range of motion; it was only displayed in the short term. No studies demonstrated increased complications, including venous thromboembolism [88–91] or the need for transfusions [88–91], with the administration of TXA in a PAI.

Exclusion criteria for the studies listed include age < 18 [92] or > 80 [91], allergy to TXA [89, 91], secondary osteoarthritis [88, 89], bilateral TKA [88], cruciate-retaining prostheses [88], renal dysfunction [88, 91, 92], ischemic heart disease [88, 89], hepatic disease [88], malignancy [90], respiratory disease [88, 91], cerebrovascular disease [89], subarachnoid hemorrhage [89], acquired color-blindness [89], coagulopathy [88–91], or anticoagulation [89, 90], thrombocytopenia [88, 91], history of a pro-thrombotic condition or previous venous thromboembolism [88, 89, 91], pregnancy [91], breastfeeding [91], donated preoperative autologous blood [91, 92], postoperative allogenic blood transfusion [92], use of an unexpected prosthesis [92], severe synovectomy during the procedure [92] or low preoperative hemoglobin [89, 91]. Special consideration should be given to patients in these categories before providing TXA in a periarticular infiltration.

## Conclusion

The ideal cocktail for pain control has not been established yet. Multiple drugs can be administered safely in this “cocktail” to help with pain control following a total knee and hip replacement. The medications should be individualized to avoid administering the medications to high-risk patients. Risk factors should be weighed against the benefits of the medications included in the periarticular injection. Even though the surgeon is administering the drug, there should be communication with the other team members (anesthesiologist, Internal medicine, ward physician, and pharmacists) to collaborate regarding the medications used and dosages. Certainly, other drugs might also prove beneficial in the future to optimize periarticular injections, and hopefully, future research might identify the optimal combination. The senior author uses periarticular injections around total knee and total hip replacements consistently with great success and have done so for the past 5 years. During this time, the “cocktail” has evolved and currently consists of 0.5% Ropivicaine 200 mg, Epinephrine 0.3 ml (1:1000), Ketorolac 30 mg, Dexmedetomidine 50ug, Methylprednisolone 40 mg and 1 g of Tranexamic acid. The senior author tries to adjust the “cocktail” if certain risk factors are present. That includes omitting Dexmedetomidine if patients have a history of bradycardia, hypotension etc., or Methylprednisolone if patients are a higher risk for infections among other things or adjusting the dosage of ropivacaine if a peripheral block was performed by the anesthesiologist. The main injection points utilized in total hip and knee replacements are at the highest concentrations of the nociceptors as described above.

## Data Availability

Not applicable – review article.
